# Cellular, Molecular and Biochemical Impacts of Silver Nanoparticles on Rat Cerebellar Cortex

**DOI:** 10.3390/cells10010007

**Published:** 2020-12-22

**Authors:** Eman M. Mohamed, Asmaa A. A. Kattaia, Rehab S. Abdul-Maksoud, Samia A. Abd El-Baset

**Affiliations:** 1 Department of Medical Histology and Cell Biology, Faculty of Human Medicine, Zagazig University, Zagazig 44519, Egypt; emanmosallam79@gmail.com (E.M.M.); drsamia2013@yahoo.com (S.A.A.E.-B.); 2 Department of Medical Biochemistry, Faculty of Human Medicine, Zagazig University, Zagazig 44519, Egypt; rehabshaaban2014@gmail.com

**Keywords:** cerebellar cortex, impacts, rat, silver nanoparticles

## Abstract

Background: The excessive exposure to silver nanoparticles (Ag-NPs) has raised concerns about their possible risks to the human health. The brain is a highly vulnerable organ to nano-silver harmfulness. The aim of this work was to evaluate the impacts of Ag-NPs exposure on the cerebellar cortex of rats. Methods: Rats were assigned to: Control, vehicle control and Ag-NP-exposed groups (at doses of 10 mg and 30 mg/kg/day). Samples were processed for light and electron microscopy examinations. Immunohistochemical localization of c-Jun N-terminal kinase (JNK), nuclear factor kappa beta (NF-κB) and calbindin D28k (CB) proteins was performed. Analyses of expression of DNA damage inducible transcript 4 (*Ddit4*), flavin containing monooxygenase 2 (*FMO2*) and thioredoxin-interacting protein (*Txnip*) genes were done. Serum levels of inflammatory cytokines were also measured. Results: Ag-NPs enhanced apoptosis as evident by upregulation of *Ddit4* gene expressions and JNK protein immune expressions. Alterations of redox homeostasis were verified by enhancement of *Txnip* and *FMO2* gene expressions, favoring the activation of inflammatory responses by increasing NF-κB protein immune expressions and serum inflammatory mediator levels. Another cytotoxic effect was the reduction of immune expressions of the calcium regulator CB. Conclusion: Ag-NPs exposure provoked biochemical, cellular and molecular changes of rat cerebellar cortex in a dose-dependent manner.

## 1. Introduction

The innovation of nanomaterials has evoked advancements in science and engineering research. Nanomaterials are considered the materials for future owing to their distinctive properties and varied applications [1]. Silver nanoparticles (Ag-NPs) are among the most hazardous metal nanoparticles due to the extensive uses and the inevitable exposure of human. They have been widely used in the production of cosmetics, healthcare products and wound dressings. They have antibacterial efficacy and more recently, synergistic effects with antibiotics against resistant bacterial species [2]. The anti-fungal properties against Candida albicans and the antiviral potentials against SARS-Cov (severe acute respiratory syndrome associated coronavirus), influenza A/H1N1 (influenza A virus subtype H1N1), HIV (human immunodeficiency virus), HBV (hepatitis B virus) and encephalitis viruses have been also reported [3,4] Ag-NPs are widely used in areas of biotechnology such as nanowires and nanotubes [5]. The impact of Ag-NPs depends mainly on the particle size. The smaller particles can induce greater harmfulness due to increased mass diffusivity, attachment efficiency and deposition velocity of NPs over the biological or solid surfaces [6]. However, the shape and solubility of Ag-NPs could also affect the cellular uptake, which in turn influence the cytotoxicity [7].

Cellular uptake of Ag-NPs occurs via active and passive transport; it includes phagocytosis, endocytosis, diffusion or direct penetration through the ion channel [8,9]. When Ag-NPs enter cells; they become more toxic than in the environment. Inside cells; nano-silver are converted from the elemental silver (Ag^0^), to Ag^+^ ions then to silver oxide species (Ag–O and, lastly, to silver sulfide species (Ag–S) that bind to thiols [10] However, in the environment, Ag-NPs enter sulfidation reactions to Ag_2_S, which decrease their toxicity [11].

Nano-silver can be absorbed through different routes as ingestion, injection, inhalation or skin contact. When enter blood stream; it binds to plasma proteins and blood cells to be distributed to all the organs [12]. Brain is liable to silver accumulation comparative to other organs [13]. They can reach the brain through the upper respiratory tract and sensory nerves in the olfactory bulb [14] or through the blood brain barrier (BBB) by transcytosis of capillary endothelial cells [15]. They can cross the tight junction of BBB and increase its permeability favoring many other harmful substances to enter the brain [16]. Additionally, they can pass through synaptic membrane or neuron cell membrane through the ionic channels for Na^+^, K^+^, Ca^2+^ and Cl^−^ [17].

By reviewing previous studies, Ag-NPs accumulate in the brain tissues after exposure through different routes [13]. Different particle size and coating can produce distinct patterns of neurotoxicity [18]. Rahman et al. studied the acute toxicity of Ag-25 nm nanoparticles administrated at high doses (100 mg/kg, 500 mg/kg and 1000 mg/kg body weight) intraperitoneally. Their results revealed altered expression of genes involved in oxidative stress and apoptosis in regions of the brain from caudate nucleus, frontal cortex and hippocampus [19]. Liu and his colleagues investigated the effects of Ag-NPs (at doses of 3 and 30 mg/kg body weight-average size 244.5) on rat hippocampus following nasal administration. They reported abnormal spatial cognition and over-generation of reactive oxygen species (ROS) in hippocampal homogenates [20]. Xu et al. showed that oral Ag-NPs (1 and 10 mg/kg body weight-10 nm in diameter) were able to cross the BBB and cause pathological changes in the hippocampus [21]. Hadrup et al. reported that the concentrations of brain neurotransmitters were altered following 28 days of oral administration of 14 nm Ag-NPs or ionic silver to rats [22]. Park et al. used Ag-NPs of similar sizes to our study (22 nm, 42 nm, 71 nm and 323 nm) at a dose of 1 mg/kg body weight for 14 days, however, no data on the histological changes of the brain were evaluated [23]. Ag-NPs (at doses of 0.2 and 1mg/kg body weight-21.7 to 24.4nm in diameter-administered as nasal drops) induced apoptosis of rat cerebellar granule cells mediated by a caspase-dependent signaling pathway [24].

We analyzed different genes and markers involved in apoptotic, inflammatory and oxidative stress cascades. DNA damage inducible transcript 4 gene (*Ddit4*, also known as Redd1 and Dig-2) is a stress responsive gene induced following DNA damage and has a key role in the control of apoptosis and inflammation. *Ddit4* expression increased in neurodegenerative diseases such as Alzheimer and Parkinson disease and is associated with mitochondrial dysfunction and neuronal cell death [25]. c-Jun N-terminal kinase (JNK) is one of the mitogen-activated protein kinases (MAPK); a group of serine-threonine proteins responsible for modulating many cellular responses. Under stress conditions, JNKs stimulate apoptosis by enhancing pro-apoptotic genes or by affecting the actions of pro- and anti-apoptotic proteins of mitochondria [26]. Thioredoxin-interacting protein (*Txnip*) is a redox-sensitive gene involved in multiple pathways that connect oxidative/glucose stress and inflammation to cellular damage. *Txnip* is considered a promising target for new brain therapies [27]. Flavin containing monooxygenase 2 (*FMO2*) gene is implicated in the oxidative metabolism of different xenobiotics. *FMO2* catalyzes the oxidation of reduced glutathione (GSH) and its overexpression can disturb the oxidized glutathione oxidized glutathione (GSSG)/GSH balance [28]. Nuclear factor kappa beta (NF-κB) protein acts as a crucial inflammatory mediator that influence the expression of several genes involved in inflammation, cancer and oxidative stress. NF-κB contributes to mitochondrial dysfunction; hence, it affects nervous system function [29]. Calbindin D28k (CB) is a calcium-binding protein that plays a neuroprotective role by buffering intracellular Ca^2+^. CB has been involved in integrative functions of the cerebellar cortex [30]. CB is used as a marker for Purkinje cells in normal and degenerative conditions [31]. CB depletion is combined with serious neurological disorders involving motor sensory, cognitive and affective impairments [32].

Previous studies reported the adverse effects of Ag-NPs on different parts of the brain especially hippocampus. However, few in-vivo studies have focused on the cerebellar cortex especially the histopathological changes at ultrastructural level. Based on this background, the aim of the current study was to assess the impacts of Ag-NPs exposure on the cerebellar cortex of adult male albino rats especially changes at the ultrastructural level. We also investigated changes at the biochemical and molecular levels.

## 2. Materials and Methods

### 2.1. Chemicals

Silver nanoparticles Ag-NPs (nano powder, CAS-No. 7440-22-4) were obtained from Sigma-Aldrich Chemicals, Cairo, Egypt.

They contain poly vinyl pyrrolidone (PVP) as a dispersant. They have the following properties: Purity 99.5%; the hydrodynamic diameter after water dispersion ranges from 45 to 120 nm, average 83 nm (SD 37), D10 45 nm, D50 76 nm and D90 120nm based on nanoparticle aracking Analysis, formula weight 107.87 g/mole; negative charge with a zeta potential −33 mV, as provided by the supplier.

PVP (CAS-No. 9003-39-8; powder) was obtained from Sigma-Aldrich Chemicals, Cairo, Egypt.

### 2.2. Experimental Animals

Forty Wistar albino rats (adult 7 to 9-week age, male, weighing 200–250 g) were attained from the Animal House of the Faculty of Medicine, Zagazig University, Egypt. We put the animals in plastic cages under normal laboratory conditions with suitable temperature (22 ± 2 °C), humidity (60 ± 10%) and organized photoperiod of 12 h-dark and 12 h-light. They were allowed free access to food and water. All procedures were done according to institutional guidelines for the use of experimental animals and approved by Institutional Animal Care and Use Committee (IACUC) (protocol approval number: 6937), Zagazig University, Egypt. Procedures were conformed to NIH (National Institutes of Health) Guidelines for the Care and Use of Laboratory Animals.

### 2.3. Characterization of Ag-NPs

The size and shape of Ag-NPs were inspected using transmission electron microscopy (JEOL JEM 1010; Jeol Ltd., Tokyo, Japan). The aqueous dispersion of the nanoparticles was dropped on a carbon-coated copper grid which was dried then examined.

### 2.4. Experimental Design

After 1-week of acclimation, rats were parted randomly into four groups (10 rats each). Treatments were given by oral gavage for 28 days. Group I (control group) received saline in an equivalent volume to that in Ag-NPs-treated groups. Group II (vehicle control group) received PVP (11.5 mg/mL) [22]. Group III (low-dose group) and group IV (high-dose group) were administered Ag-NPs at concentrations of 10mg and 30mg/kg/day, respectively (dissolved in saline solution, gavage volume 10 mL/kg) [23,33]. We used the oral route for Ag-NPs administration as it is considered a typical environmental exposure from contaminated water, food, medications and cosmetics [21].

**Figure 1:**
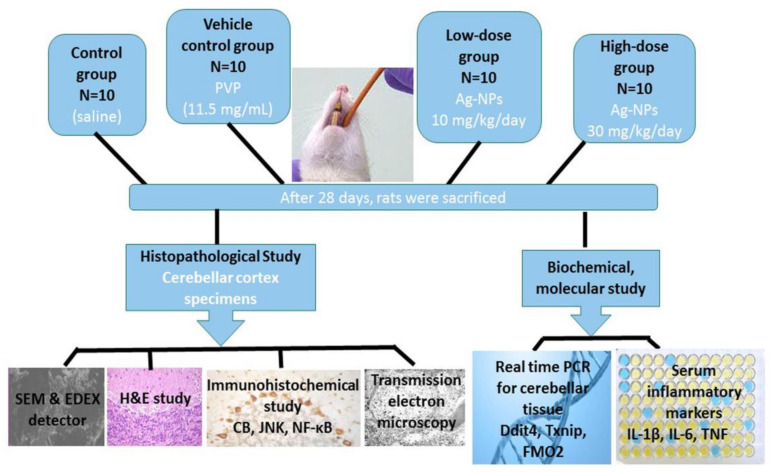
Experimental design and methods of study. Rats were parted randomly into four groups (10 rats each). Group I (control group) received saline in an equivalent volume to that in silver nanoparticles (Ag-NPs)-treated groups. Group II (vehicle control group) received poly vinyl pyrrolidone (PVP) (11.5 mg/mL). Group III (low-dose group) was administered Ag-NPs at a dose of 10mg/kg/day and group IV (high-dose group) was administered Ag-NPs at a dose of 30mg/kg/day. Treatments were given by oral gavage for 28 days. Cerebellar specimens were processed for histopathological preparations and molecular analysis. Blood samples were taken for biochemical analysis.

At the end of experiment: Rats were injected with intraperitoneal thiopental 50 mg/kg. Cerebellar specimens were cut; parts of them were used for histopathological preparations; and others were frozen immediately and stored at –80 °C until preparing tissue homogenates for molecular analysis (Figure 1).

### 2.5. Biochemical and Molecular Study

#### 2.5.1. Measurement of Inflammatory Markers

Blood samples were left to clot for 2 h and then centrifugation was conducted at 1000× *g* for 20 min to separate sera which were stored at −20 °C till being used. Assays of serum interleukin-1 beta (IL-1β), interleukin-6 (IL-6) and tumor necrosis factor-alpha (TNF-α) were achieved using commercially available enzyme linked immunosorbent assay (ELISA) kits (R&D Systems, Minneapolis, MN, USA) conferring to manufacturer’s guidelines.

#### 2.5.2. RNA Extraction and Quantitative Real Time Transcription Polymerase Chain Reaction (Real-Time PCR)

Extraction of total RNA from the cerebellar tissue was achieved using Trizol reagent (Invitrogen, Carlsbad, CA 92008, USA) according to the instructions of the manufacturer. RNA concentration and purity were estimated using spectrophotometer (NanoDrop ND-1000, Wilmington, DE 19810, USA) at 260 and 280 nm, respectively. Reverse transcription was performed using Superscript II reverse transcriptase kit (Invitrogen, Thermo Fisher Scientific) according to the manufacturer’s protocol.

Gene expression analyses of *Ddit4*, *Txnip* and *FMO2* were performed in duplicate using real-time PCR detection system (Light Cycler, Roche Diagnostics, Rotkreuz, Switzerland). Glyceraldehyde-3-phosphate dehydrogenase (*GAPDH*) was used as a house keeping gene for gene expression normalization. PCR amplification was performed in 20 µL reaction mixture containing 1 µL template cDNA, 0.4 mM of each primer, 10 µL of SYBR green PCR master (Qiagen, Hilden, Germany) and the final volume was adjusted with double distilled water. The sequence of the used primers is listed in (Table 1). The following cycling conditions were used: Initially 10 min at 94 °C, then 35 cycles of denaturation for 10 s at 94 °C, annealing, extension for 18 s at 72 °C and, finally, at 72 °C for 10 min. Melting curves were constructed to ensure amplification of the specified genes. Gene expression of the specified genes was represented as fold change which was calculated by the 2^ΔΔCT^ method [34].

### 2.6. Histopathological Study

#### 2.6.1. Scanning Electron Microscopy (SEM) and Energy Dispersive X-Ray (EDX) Detector

Detection of Ag-NPs in the cerebellar cortex of exposure groups was done using SEM (JEOL JSM-6510LV electron microscopy; Jeol Ltd., Tokyo, Japan) and EDX detection by X-ray analyzer (X-Max^N^ 20 SDD system, Oxford Instruments, Oxford, UK). In this technique, a high-resolution image was generated by scanning the prepared sample, and then EDX detector verifies the elemental compositions of the image.

#### 2.6.2. Hematoxylin and Eosin (H&E) Study

Specimens for light microscopy were fixed in 10% buffered formalin and processed to prepare 5-µm-thick paraffin sections for H&E stain [35].

#### 2.6.3. Immunohistochemical Study

Following the manufacturer’s instructions, avidin biotin complex (ABC) method (ABC Peroxidase Staining Kits, Code No. 32020, Thermo Scientific, Rockford, IL, USA) was used for immunohistochemical staining of the calcium binding protein CB, the apoptotic marker JNK and the inflammatory marker NF-κB. Removal of wax and hydration of sections of paraffin were the beginning points. Antigen retrieval was performed by using citrate buffer and microwave for 15 min. Tissues block was done by bovine serum albumin. Then, sections were incubated with the specific primary antibody overnight (4 °C): Anti-CbD28k (rabbit polyclonal antibody; Cat. #PA5-85669; dilution 1/500; Thermo Scientific, San Jose, CA, USA), anti-JNK antibody (rabbit polyclonal antibody; code No. ab112501; dilution 1/100; Abcam, Cambridge, UK) and anti-NF-κB (rabbit polyclonal antibody; Cat. #RB-9034-R7; dilution 1/100; Thermo Scientific, San Jose, CA, USA). Recognition was accomplished by secondary antibodies and labeled horseradish peroxidase followed by colorimetric detection by 3, 30-diaminobenzidine (DAB). Hematoxylin was used as a counterstain. Negative control slides were put in phosphate-buffered saline as a replacement for the primary antibody. Under light microscopy, the brown color indicated the antigensite [36].

#### 2.6.4. Transmission Electron Microscopy (TEM) Study

Fixation of the specimens was done by phosphate-buffered glutaraldehyde (pH 7.4), and post fixation by 1% osmium tetroxide at 4 °C. The specimens then dehydrated and embedded in epoxy resin. Cutting by Leica ultra-cut (UCT) and staining by uranyl acetate and lead citrate were performed [37]. Ultrathin sections (50 nm thick) were checked and photographed using TEM (JEOL JEM 1010; Jeol Ltd., Tokyo, Japan) in the Regional Center of Mycology and Biotechnology (RCMB), Al-Azhar University, Egypt.

### 2.7. Morphometric Study

The data were investigated by Leica QWin 500 software using digital camera linked to an optical microscopy (Olympus, Tokyo, Japan). Positive brown cells were counted in anti-CbD28k, anti-JNK and anti-NF-κB immune-stained sections. In 7286, 78 µm^2^ measuring frames at a magnification of 400×, ten non-overlapping fields from each rat were randomly selected and investigated by analyst who was unaware of the experiment.

The linear density of Purkinje cells was also measured in H&E stained sections. For each rat, Purkinje cells (recognized by nerve cell body) were counted in each of 10 intact cerebellar lobules of sagittal sections at 200× magnification, and then the mean value for each section was estimated. The density was calculated as the mean value of cell count per millimeter length of cerebellar tissue [38].

### 2.8. Statistical Analysis

Statistical Package for Social Sciences (SPSS) version 22.0 (IBM Corp., Armonk, NY, USA) was used to analyze the data. Values were expressed as mean ± standard deviation (X ± SD). ANOVA test followed by Tukey’s post-hoc test was used. The probability values (*p*) less than 0.05 were regarded as significant and highly significant with *p* values less than 0.001.

## 3. Results

### 3.1. Characterization of Ag-NPs

**Figure 2:**
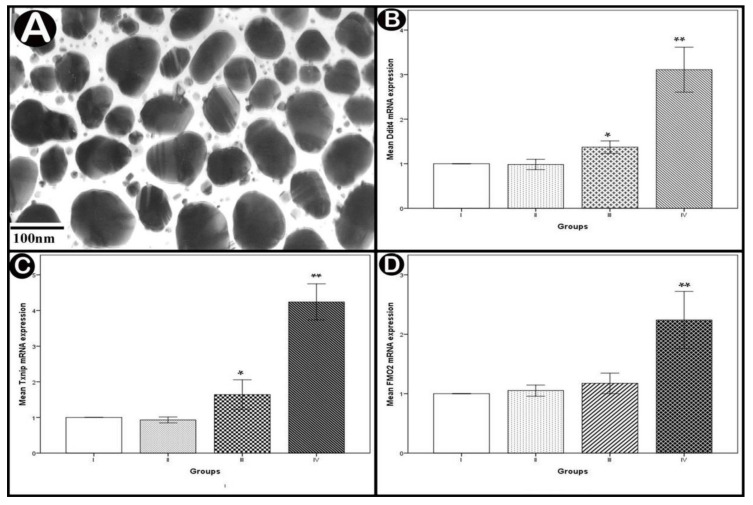
(**A**) TEM image showing average diameters of Ag-NPs. (**B**–**D**) real-time PCR analysis of mRNA expressions of DNA damage inducible transcript 4 (*Ddit4*), thioredoxin-interacting protein (*Txnip*) and flavin containing monooxygenase 2 (*FMO2*) in the cerebellar cortex. Values are expressed as mean ± standard deviation (X ± SD); *: Significant difference (*p* < 0.05) and **: Highly significant difference *(p* < 0.001); *n* = 10 animals.

They appeared spherical or semi-spherical with diameters ranges from 45 to 120 nm (average 83 ± 37 nm) (Figure 2A). 

### 3.2. Biochemical and Molecular Results

#### 3.2.1. Measurements of Serum Proinflammatory Cytokines

Measurements of IL-1β and TNF-α demonstrated significant increases in the low-dose group (*p* < 0.05) and highly significant increases in the high-dose group (*p* < 0.001) when compared to the control group. There was a non-statistically significant rise in IL-6 in the low-dose group (*p* > 0.05), while there was a highly significant increase in the high-dose group (*p* < 0.001) compared to the control (Table 2).

#### 3.2.2. Real-Time PCR Analysis of Apoptotic and Oxidative Stress Genes

Statistical analysis of the mean *Ddit4* and *Txnip* mRNA levels showed significant increases in the low-dose group (*p* < 0.05) and highly significant increases in the high-dose group (*p* < 0.001) compared to normal controls. The increase was non-statistically significant regarding the mean *FMO2* mRNA level in the low-dose group (*p* > 0.05) but highly significant in the high-dose group (*p* < 0.001) in comparison with normal controls (Figure 2B–D).

### 3.3. Histopathological Results

The control groups I (control) and II (vehicle control) exhibited almost similar results. Accordingly, only group I results were presented in figures.

#### 3.3.1. SEM and EDX Analysis Results

**Figure 3:**
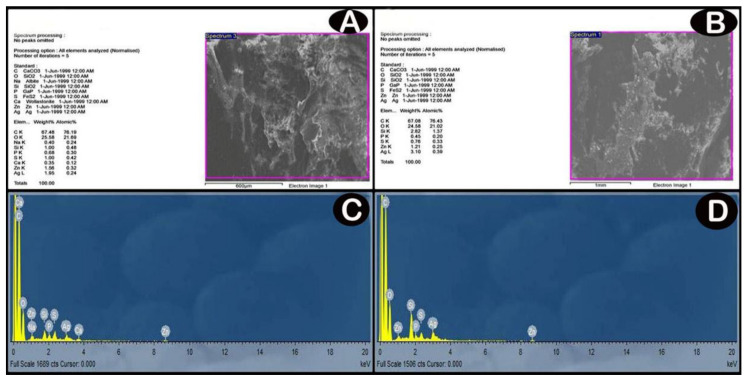
(**A**,**B**) SEM images; backscattered electron images show contrast due to different element compositions for low-dose and high dose groups, respectively. (**C**,**D**) analyses of EDX for detection of these elements and their relative amounts (atomic % and weight %) by the production of an X-ray spectrum from the whole scan area of the SEM. The *Y*-axis represents the sums of X-rays and the *X*-axis depicts their energy level (KeV). The elements are identified by the place of the peaks, while the height of the peaks is used to measure the quantity of each element. (**C**) low-dose group EDX spectrum shows a significant amount of Ag (one prominent peak at 3 KeV). (**D**) high-dose group EDX spectrum shows increased the amount of Ag compared with low-dose group (one prominent peak at 3 KeV).

SEM image and EDX spectrum analyses indicated the presence of considerable amounts of Ag-NPs in the cerebellar cortex, which increased in a dose-dependent manner in the exposure groups (Figure 3).

#### 3.3.2. H&E Results

**Figure 4:**
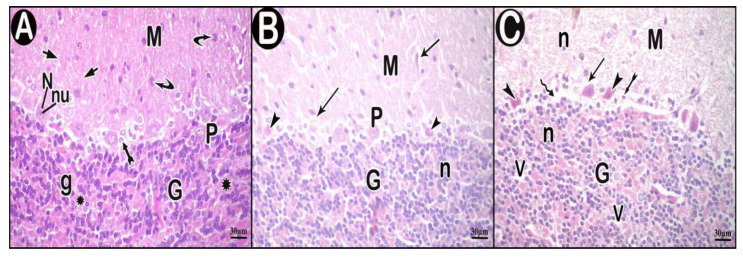
Hematoxylin and eosin (H&E)-stained sections in cerebellar cortex of the study groups. (**A**) control group. (**B**) low-dose group. (**C**) high-dose group. Outer molecular layer (M), fibers (short arrow), scattered cells (curved arrow), normal Purkinje cells (P), Bergmann astrocytes (tailed arrow), nuclei of Purkinje cells (N), nucleoli of Purkinje cells (nu), granular layer (G), granule cells (g), non-cellular cerebellar islands (asterisk), vacuolar spaces (arrow), atrophied shrunken Purkinje cells (arrow head), cells having darkly stained nuclei (n), areas of focal neuron loss (zigzag arrow) and vacuolations (V).

Light microscopy examination of H&E-stained sections of the control group showed that the outer molecular layer was formed of fibers mainly with few scattered cells. The middle layer contained one row of flask-shaped Purkinje cells and Bergmann astrocytes. Purkinje cells had rounded vesicular nuclei with prominent nucleoli. Granular layer had small closely packed cells together with non-cellular cerebellar islands (Figure 4A). In low-dose group, the molecular layer showed some spaces. Some Purkinje cells appeared normal while others appeared atrophied, shrunken with ill-defined nuclei and surrounded by vacuolar spaces. Some cells of the granular layer appeared with darkly stained nuclei (Figure 4B). In high-dose group, the molecular layer contained many vacuolar spaces and pyknotic nuclei. Purkinje cells appeared distorted, shrunken with pyknotic ill-defined nuclei and acidophilic cytoplasm, and surrounded by vacuolar spaces. Areas of focal loss in neurons were also seen. Bergman astrocytes appeared swollen with dark nuclei and wide perinuclear spaces. Some granular layer cells had darkly stained nuclei with spaces in between. Cerebellar islands showed vacuolations (Figure 4C).

#### 3.3.3. Immunohistochemical Results

**Figure 5:**
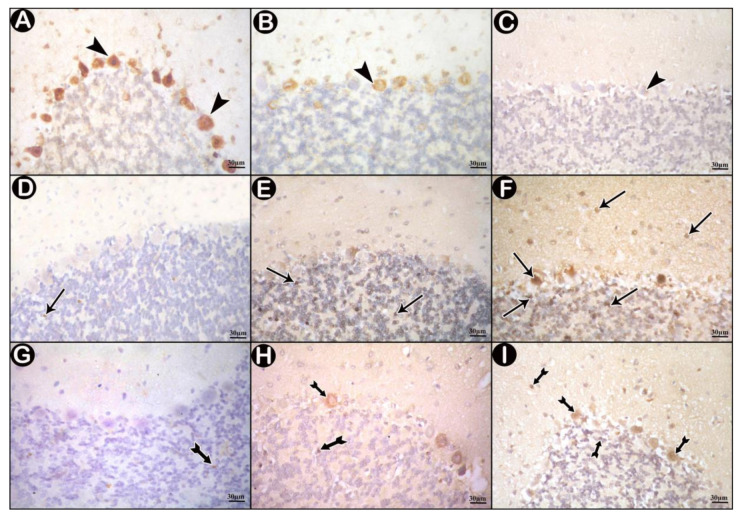
Immunohistochemically stained sections in cerebellar cortex of the study groups. (**A**,**D**,**G**) control group. (**B**,**E**,**H**) low-dose group. (**C**,**F**,**I**) high-dose group. (**A**–**C**) immune reactions for Calbindin D28k (CB) (arrow head). (**D**–**F**) c-Jun N-terminal kinase (JNK) immune-reactions (arrows). (**G**–**I**) nuclear factor kappa beta (NF-κB) immune reactions (tailed arrows).

Immunohistochemically stained sections for CB of control group showed positive cytoplasmic immune reaction in most of Purkinje cells (Figure 5A), few immune reactive cells were detected in low-dose group (Figure 5B), while the reactions were nearly absent in high-dose group (Figure 5C). 

Control sections stained for JNK revealed scanty positive immune reactions (Figure 5D). Some positive immunoreactions were detected in the granular layer of low-dose group (Figure 5E). In high-dose group, many immune reactions were seen all over the three layers of the cerebellar cortex (Figure 5F).

NF-κB-stained sections showed very few immune reactions in control group (Figure 5G). In low- dose group, brown immune reactions in some cells were seen (Figure 5H), and several positive cells appeared in high-dose group (Figure 5I).

#### 3.3.4. Ultrastructure Results

**Figure 6:**
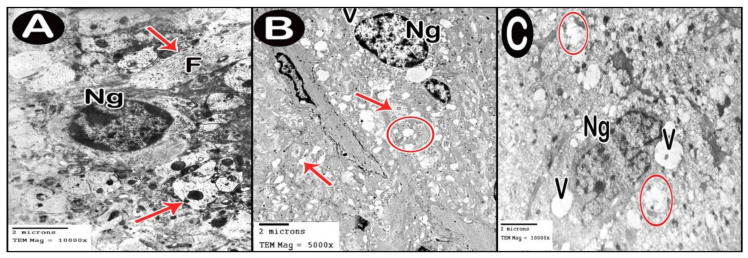
Electron micrographs of cerebellar cortex molecular layer. (**A**) control group. (**B**) low-dose group. (**C**) high-dose group. Axons (arrow), neurofilaments (F), neuroglia (Ng), vacuolated neuropil (circle) and vacuoles (V).

**Figure 7:**
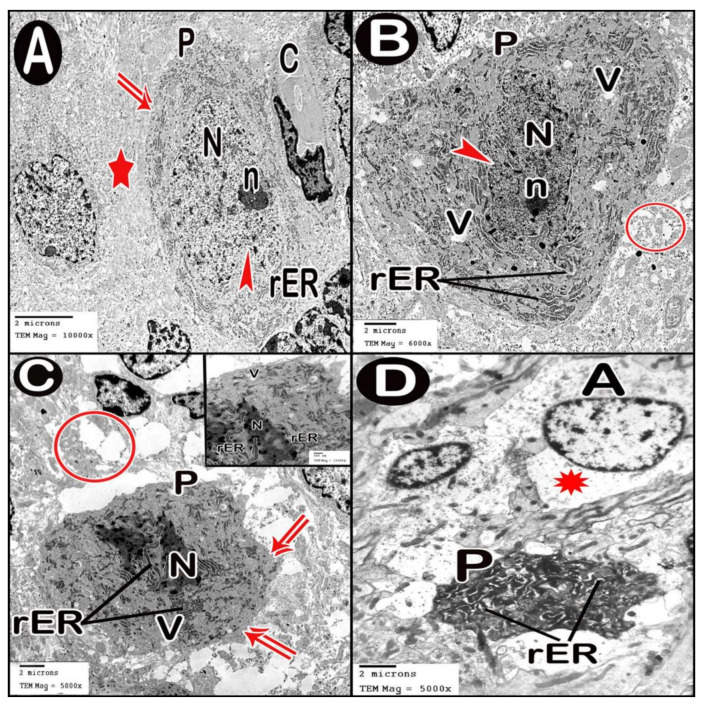
Electron micrographs of Purkinje cell layer. (**A**) control group. (**B**) low-dose group. (**C**,**D**) high-dose group. Purkinje cell (P), contour of cell membrane (double arrow), tight neuropil (star), blood capillary (C), nucleus (N) with indentation (arrow head), nucleolus (n), short profiles of rough endoplasmic reticulum cisternae (rER), swollen astrocytes (A) and loss of cytoplasmic organization (asterisk).

**Figure 8:**
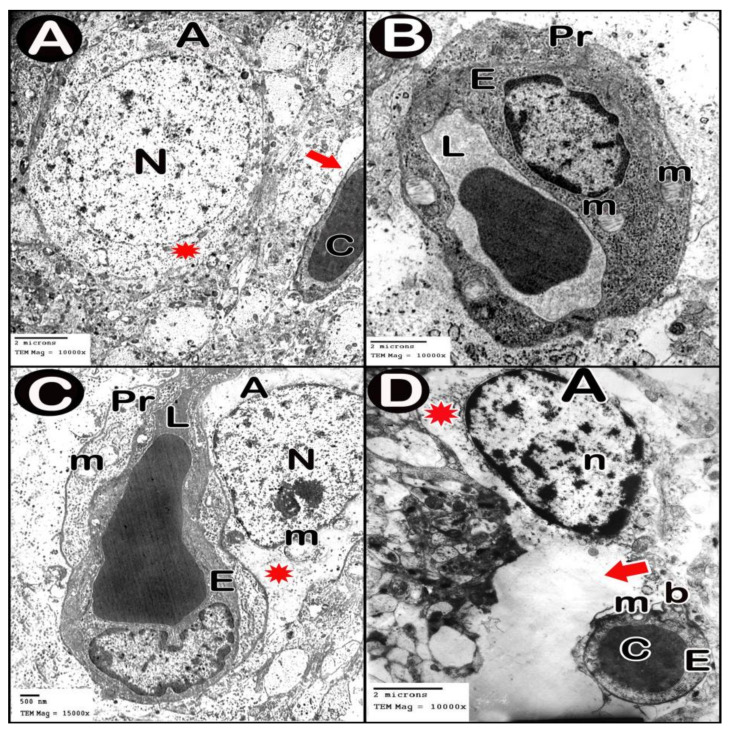
Electron micrographs of Blood brain barrier. (**A**,**B**) control group. (**C**) low-dose group. (**D**) high-dose group. Astrocyte (A), nucleus (N), cytoplasm (asterisk), blood capillary (C) separated by perivascular spaces (thick arrow), lumen of blood capillary (L), endothelial lining (E), mitochondria (m), pericyte cytoplasm (Pr), clumps of heterochromatin within and around the edge of the nucleus (n) and separation of the basement membrane (b).

**Figure 9:**
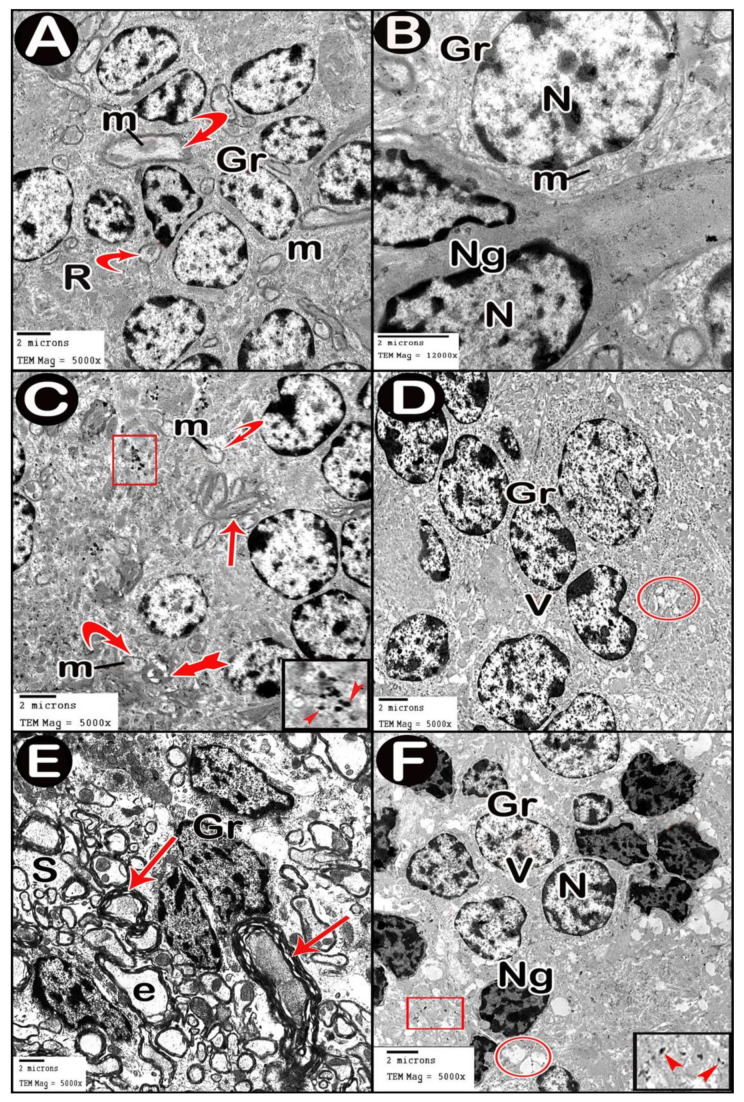
Electron micrographs of granular layer. (**A**,**B**) control group. (**C**,**D**) low-dose group. (**E**,**F**) high-dose group. Granule cells (Gr), mitochondria (m), mossy rosettes (R), neuroglia (Ng), normal myelinated fibers (curved arrow), disrupted myelin (tailed arrow), splitting of myelin sheaths (arrow), vacuoles (V), vacuolated neuropil (circle), ballooned or empty fibers (e) and wide spaces between the axons (S). Insets in C and F, magnifications of the boxed parts showing electron dense silver nanoparticles (arrow heads).

TEM examination of the cerebellar cortex of control group revealed that the molecular layer was formed of compact full neuropil (dendrites, unmyelinated axons and processes of neuroglia) and neuroglia in-between (Figure 6A). Purkinje cells appeared with regular contour, euchromatic indented nuclei, prominent nucleoli and clear cytoplasm with short profiles of rough endoplasmic reticulum cisternae, mitochondria and free ribosomes. They were surrounded by tight neuropil and blood capillaries (Figure 7A). Astrocytes had euchromatic nuclei and surrounded by a shell of cytoplasm filled with organelles. Adjacent blood capillaries were seen separated by narrow perivascular spaces. They had regular lumen, smooth endothelial lining and mitochondria with intact cristae in the cytoplasm of endothelium and pericytes (Figure 8A,B). The granular layer had well-defined ring-shaped cerebellar islands that contained myelinated fibers with spherical mitochondria, and mossy rosettes with many mitochondria. The granule cells had rounded euchromatic nuclei with peripheral clumps of heterochromatin surrounded by a shell of cytoplasm containing mitochondria, strands of rough endoplasmic reticulum and free ribosomes. In the vicinity, neuroglia had darker nuclei with marginal heterochromatin and electron dense cytoplasm (Figure 9A,B). Low-dose group ultrathin sections showed the molecular layer with some areas of vacuolated neuropil, vacuoles within some axons and neuroglia (Figure 6B). Purkinje cells showed darker nuclei, prominent nucleoli and dimples in the nuclear envelope, some dilated cisternae of rough endoplasmic reticulum and some vacuoles in the cytoplasm. The cells were surrounded by loose vacuolar spaces (Figure 7B). Perivascular astrocytes showed increased translucence of their cytoplasm. Mitochondria with ruptured cristae were found in the cytoplasm of astrocytes, capillary endothelium and pericytes (Figure 8C). In the granular layer, some nerve fibers showed disruption or splitting of myelin sheaths. Granule neurons appeared with increased condensation of nuclear chromatin, some vacuoles in the perikaryon and some areas of vacuolated neuropil between the cells. Electron dense silver nanoparticles could be detected within the neuropil (Figure 9C,D).

In ultrathin sections of high-dose group, most of the neuropil of the molecular layer appeared vacuolated and vacant; the neuroglia had many large vacuolar spaces (Figure 6C). Purkinje neurons showed irregular cell membrane and a shadow of muddy ill-defined nucleus. The cytoplasm contained dilated cisternae of rough endoplasmic reticulum and vacuoles. The cell was surrounded by vacuolated neuropil. Fragments of Purkinje cells perikaryon appeared electron dense with dilated rough endoplasmic reticulum cisternae (Figure 7C,D). Swollen astrocytes showed clumps of heterochromatin within and around the edge of the nuclei, increased matrix translucence and loss of cytoplasmic organelles. Blood capillaries were surrounded by a large perivascular space. The lining endothelium showed mitochondria with ruptured crista and separation of the basement membrane (Figure 8D). Granular layer showed irregularly arranged neurons with heterochromatic nuclei and intracellular vacuolar spaces. Cerebellar islands were distorted. Many fibers showed splitting of myelin sheath and some appeared ballooned or empty with wide spaces in-between. Adjacent neuroglia appeared with heterochromatic nuclei and surrounded by vacuolated neuropil. Electron dense silver nanoparticles could be detected within the neuropil (Figure 9E,F).

### 3.4. Morphometric Results

Statistically analyzed results of numbers of CB, JNK and NF-κB in immune-stained sections and Purkinje cell linear density in H&E sections were represented in (Table 3).

## 4. Discussion

The excessive applications of nano-silver cause wide environmental contamination and raise the hazards of human exposure [2]. The brain is a highly vulnerable organ to silver toxicity due to the prolonged exposure caused by the long biological half-life of silver in the CNS when compared with other organs [17]. It was found that silver was removed from most organs, except brain and testis after 8 weeks in rats exposed to oral Ag-NPs for 28 days [13]. The existence of BBB could decrease the rate of Ag-NPs clearance from the brain that leads to long standing adverse effects in brain tissue [39].

Ag-NPs cause tissue damage due to direct deposition in the tissues owing to their small size. Furthermore, they release large amount of free toxic silver ions (Ag^+^) [40]. Neurons are more sensitive to nano-silver because of their high metabolic requirements [41]. On comparing particle sizes; the smaller the size, the more severe adverse effects occur [42].

Adverse effects of Ag-NPs on different parts of the brain, especially hippocampus, were investigated. However, few in-vivo histopathological studies have focused on the cerebellar cortex especially at the ultrastructural level. In the current study, Ag-NPs exposure severely disrupted the architecture of the cerebellar cortex. The disruption was more apparent in the high-dose group compared with the low-dose group. Purkinje and granule neurons displayed marked alterations. The cytoplasm of the degenerated Purkinje neurons appeared deeply acidophilic which was known as “eosinophilic neuron degeneration” or “red dead neurons”. Vacuolated neuropils might result from degeneration and shrinkage of Purkinje cells with retraction of their processes leaving empty spaces. Others explained vacuolations by swelling of processes of degenerated neurons or activated glial cells [43]. Areas of focal loss in neurons were also seen. This was confirmed by the morphometric measurement of the linear density of Purkinje cells that revealed a significant and a highly significant reduction in the low-dose and high-dose group respectively compared with the control. These results were in harmony with Xu et al. [44]. Their in-vitro study showed that 20 nm Ag-NPs hinder the sprouting of neuronal outlets or elongation of axons, and produced neurotic processes degeneration. Moreover, Ag-NPs decreased cytoskeletal integrity, synaptic proteins, mitochondrial function and so cell viability.

Transmission electron microscopy pictures of treated groups of this study revealed cells having degenerated mitochondria with ruptured cristae. Similar results were shown by other studies [45]. It is well known that silver nanoparticles cause decreased ATP levels and so discomposure of cellular respiration leading to mitochondrial damage and cell death [46] Another ultrastructural finding, in the current work, was the dilated rough endoplasmic reticulum (RER) in Purkinje cells. This was in line with the findings of Zhang et al. [47] who stated that treatment of liver cells by nano-silver (≤100 nm) induced endoplasmic reticulum (ER) stress and increased the levels of chaperone proteins (ER membrane proteins that act as sensors for ER stress). The over-expression of these chaperones leads to inhibition of protein translation. Simard et al. added that this ER stress response pathway may extend to cell death [48]. We also noticed splitting of the myelin sheaths of some nerve fibers in Ag-NPs-intoxicated groups. Similarly, other researchers found myelin disintegration after 2-week-exposure to oral nano-silver (0.2 mg/kg) in rats. They concluded that oxidative stress affects the proper structure of myelin sheaths by disrupting the lipid and protein constituents of myelin membranes [49].

In the present study, TEM findings revealed that Ag-NPs induced BBB destruction as evident from, (i) astrocytes appeared swollen with loss of cytoplasmic organelles; (ii) the capillaries were surrounded by wide perivascular spaces and (iii) the lining endothelium had degenerated mitochondria. Other studies also revealed similar results in brain owed to a low dose of Ag-NP exposure by oral route [50]. Another in vitro study showed that citrate coated Ag-NPs led to brain endothelial cell membrane damage and disrupt colony formation [51]. Astrocyte revealed the appearance of nano-silver-like particles [21]. Ag+ ions trigger cell necrosis in astrocytes through disrupting the integrity of cell membrane and binding with cellular thiol groups. On the other hand, Ag-NPs stimulates apoptosis by mediating ROS production coupled with JNK activation [52]. Ag-NPs intoxication reduces the biosynthetic activities in astrocytes e.g., nerve growth factor (Nr4a1) secretion that could affect neuronal survival and protection [53]. Degeneration of glial cells causes also degeneration of cerebellar Purkinje cells [54].

Notably, we observed that Ag-NPs intake elicited different nuclear responses in light and electron microscopy slides; some nuclei seemed small and pyknotic, others appeared irregular with clumping of chromatin. These findings were in line with other investigators who reported that deposition of nano-silver in the nuclei of mesenchymal stem cells triggered DNA damage which appeared as chromatid deletions and chromatid exchanges [55]. Cell cycle arrest also happened in the G2/M phase in cells treated with high dose of nano-silver (7.5 nm). In the present work, gene expression analysis of *Ddit4* revealed that Ag-NPs exposure significantly increase *Ddit4* mRNA in a dose depending manner. *Ddit4* is believed to enhance apoptosis and decrease cell proliferation by activating caspase signaling pathway and promote autophagy by inhibiting the mammalian target of rapamycin (mTOR) activities [56]. Ag-NPs were accompanied by cellular death more than 30% [57].

In the same context, we detected a significant increase in immune histochemical expressions of the apoptotic marker JNK in Ag-NPs-exposure groups. These results conform to the results of Rinna et al. [58]. The key cytotoxic effect of Ag-NPs is apoptosis-mediated cell death [59]. Nano-silver encourages ROS generation leading to the activation of JNK and p53, and cytochrome c release which progresses to apoptosis [60]. The excess production of ROS in turn increases the pro-apoptotic kinase p38 and decreases poly ADP ribose polymerase (PARP) resulting in significant surge of caspase-3 and total p53 expressions [61]. So, nano-silver triggers both the mitochondrial and the extrinsic apoptotic pathways [22]. It was reported that granule cells are the most vulnerable cells to NPs [24,62]. JNK also stimulates the phosphorylation of the BH3 (Bcl-2 homology domain 3, proteins promote cell death) in cerebellar granule neurons promoting apoptosis by another mechanism [63]. 

Ag-NPs act as catalyst that directly produces ROS especially in the presence of oxygen. In addition, Silver itself has strong affinity for sulfur present in cellular protein [64]. The present study proposed oxidative stress as an adverse effect of Ag-NPs exposure. We recorded enhancement of *Txnip* gene expression in Ag-NPs treated groups. *Txnip*, a member of α-arrestin family, is implicated in redox sensing and induced by ER stress. Up-regulation of *Txnip* increases ROS and compromises the antioxidant capacity of the brain [65]. We also detected upregulation of *FMO2* gene expression (the gene responsible for oxidation of reduced glutathione) following Ag-NPs exposure. These changes disrupt oxidized glutathione GSSG/GSH balance and trigger neurodegeneration [19].

Regarding the inflammatory responses elicited by Ag-NPs exposure, we revealed a significant enhancement of NF-κB protein immune expressions in nano-silver treated groups. We also found statistically significant increases of the serum inflammatory cytokines; IL-1β, IL-6 and TNF-α following Ag-NPs administration in a dose-dependent manner. This could be explained by the excess release of ROS. Perrone et al. declared that *Txnip* gene up-regulation stimulates the activation of NF-κB protein and the release the pro-inflammatory mediators such as TNF-α and IL-1β [66]. In line with our results, in-vivo oral nano-silver (22, 42, 71 nm) given 1 mg/kg for 14 days elevated the serum levels of TGF-β, IL-1, IL-4, IL-6, IL-12 and increased delivery of B cells and natural killer cells [23]. Nano-silver (10 and 75 nm) motivated inflammation in vitro by stimulating NF-κB and AP1 (activator protein-1) pathways [67]. Interestingly, JNK is activated by inflammatory mediators such as IL-6 and TNF-α; activated JNK phosphorylates c-Jun (a component of AP-1 complex); AP-1 complex controls the transcription of inflammation-related genes [68] *Ddit4* overexpression also plays a principal role in the activation of inflammation independently of mTOR through stimulating the expression of NF-κB and proinflammatory cytokines and activating JNK. All these events aggravate inflammation [69].

Nano-silver increases the intracellular calcium levels in neuron-enriched culture [70]. Calcium homeostasis deterioration is one of the key mechanisms of Ag-NPs neurotoxicity [71]. When N-methyl-d-aspartate receptors (NMDAR) are over-activated by nano-silver; calcium overflows into cells, excessively taken by mitochondria causing mitochondrial degeneration and dysfunction leading to generation of ROS and apoptosis [72]. CB is a calcium-binding protein that maintains calcium homeostasis. CB plays a pivotal role in preventing neuronal death by blocking several pro-apoptotic pathways [73]. CB protects against oxidative stress and toxic agents [74]. CB level significantly diminishes during degenerative neuronal diseases [75]. In nano-silver exposed groups, we reported a significant decline in immune expressions of CB in Purkinje cells. Depletion of CB reduces the buffering capacity of neurons and causing increase of intracellular and intranuclear calcium [76].

**Figure 10:**
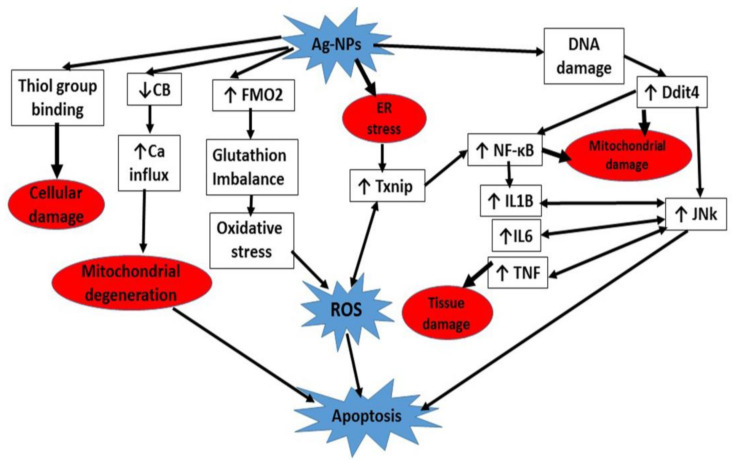
A summary of different cascades involved in Ag-NPs neurotoxicity.

As a consequence of tissue damage, the function of the cerebellum is severely compromised. Cerebellar injuries affect the motor coordination performance which is termed clinically as cerebellar ataxia. It is manifested by diminished locomotor activity in the form of decrease in the moving distance and moving velocity, failure in the proper foot placement and behavior impairment [77,78] (Figure 10).

## 5. Conclusions

Applications of Ag-NPs in water purification and biosensors, medications as nasal decongestants and oral hygiene preparations, wound dressings applied for long periods and the unlimited use of cosmetics nowadays are important risk factors of human exposure to Ag-NPs threats. Ag-NPs exposure provoked biochemical, cellular and molecular changes of rat cerebellar cortex in a dose-dependent manner. The key mechanisms included activation of apoptosis cascades coupled with stimulation of oxidative stress and inflammation pathways. This was evident by the upregulation of *Ddit4* gene expressions and JNK protein immune expressions. Alterations of redox homeostasis were verified by enhancement of *Txnip* and *FMO2* gene expressions, favoring the activation of inflammatory responses and increases in NFκB protein immune expressions and serum inflammatory mediator levels (IL-1B, IL6 and TNF-α). Another considerable cytotoxic effect of nano-silver was the reduction of CB protein immune expressions, the crucial regulator of intracellular calcium level.

## Figures and Tables

**Table 1:** Primers and annealing conditions used for real-time PCR.

Gene	5’–3’ Forward Primer	5’–3’ Reverse Primer	Annealing Condition
*Ddit4*	TAACACCAGGGAGCTGC	ACAGTTCACTCCTCCAGTACA	56 °C, 9 s
*Txnip*	GGAGAAAGTTCTGCTCTCG	AAGTGCTAAGGCGGAGTAA	56 °C, 9 s
*FMO2*	TCACCTGGAGAAGCCAAC	CGGTGATGGAGAAAAGTG	56 °C, 7 s
*GAPDH*	GTATGTCGTGGAGTCTACTG	TTTAGTGGGCCCTCGGC	58 °C, 6 s

**Table 2:** Biochemical parameters.

	Control Group	Vehicle Control Group	Low-Dose Group	High-Dose Group
IL-1β (pg/mL)	37.2 ± 7.6	35.3 ± 6.6	52.3 ± 13.0 *	123.6 ± 12.1 **
IL-6 (pg/mL)	18.4 ± 5.5	19.0 ± 4.7	38.7 ± 4.1	138.2 ± 5.7 **
TNF-α (pg/mL)	10.3 ± 1.9	11.0 ± 3.4	18.2 ± 4.5^*^	27.2 ± 10.1 **

TNF-α, tumor necrosis factor-alpha; IL-1β, interleukin-1 beta; IL-6, interleukin-6. Values are expressed as mean ± standard deviation (X ± SD); *: Significant difference (*p* < 0.05) and **: Highly significant difference *(p* < 0.001); *n* = 10 animals.

**Table 3:** Number of anti-JNK, anti-NF-κB immune-stained cells, anti-calbindin D28k (CB) and Purkinje cell linear density.

	Control Group	Vehicle Control Group	Low-Dose Group	High-Dose Group
**Anti**-**CB**	10.2 ± 2.6	10.5 ± 2.8	5.6 ± 3.0 *	1.1 ± 1.0 **
**Anti**-**JNK**	0.9 ± 0.6	1.3 ± 0.7	28.2 ± 7.7 **	103.1 ± 14.7 **
**Anti**-**NF**-**κB**	1.6 ± 1.1	1.8 ± 0.9	17.6 ± 6.0 **	36.9 ± 8.3 **
**Purkinje Cell Linear Density**	19.5 ± 3.6	20.2 ± 3.6	13.9 ± 2.6 *	8.0 ± 2.2 **

Values are expressed as mean ± standard deviation (X ± SD); *: Significant difference (*p* < 0.05); **: Highly significant difference *(p* < 0.001); *n* = 10 animals.

## Data Availability

All data generated or analysed during this study were included in this published article.

## Abbreviations

Ag-NPs	Silver nanoparticles
AP-1	Activator protein-1
BBB	Blood brain barrier
CB	Calbindin D28k
Ddit4	DNA damage inducible transcript 4
EDX detector	Energy dispersive X-ray detector
ER	Endoplasmic reticulum
FMO2	Flavin containing monooxygenase 2
GSH	Reduced glutathione; GSSG: Oxidized
GSSG	Oxidized glutathione
H&E	Hematoxylin and Eosin
IACUC	Institutional Animal Care and Use Committee
IL-1β	Interleukin-1 beta
IL-6	Interleukin-6
JNK	c-Jun N-terminal kinase
mTOR	mammalian target of rapamycin
NF-κB	Nuclear factor kappa beta
PCR	polymerase chain reaction
PVP	Poly vinyl pyrrolidone
ROS	Reactive oxygen species
SEM	Scanning electron microscopy
SPSS	Statistical Package for Social Sciences
TEM	Transmission electron microscopy
TNF-α	Tumor necrosis factor-alpha
Txnip	Thioredoxin-interacting protein
